# A genetic model of the effects of insecticide-treated bed nets on the evolution of insecticide-resistance

**DOI:** 10.1093/emph/eov019

**Published:** 2015-08-29

**Authors:** Philip L. G. Birget, Jacob C. Koella

**Affiliations:** ^1^Imperial College London, Life Sciences Division, Silwood Park, Ascot, England and; ^2^Institute of Biology, Université de Neuchâtel, 11 rue Emile-Argand, CH-2000 Neuchâtel, Switzerland; ^3^Present address: Institute of Evolutionary Biology, University of Edinburgh, Ashworth Laboratories, Scotland

**Keywords:** malaria control, insecticide-treated bed nets, repellency, insecticide-resistance

## Abstract

**Background and objectives**: The evolution of insecticide-resistance in malaria vectors is emerging as a serious challenge for the control of malaria. Modelling the spread of insecticide-resistance is an essential tool to understand the evolutionary pressures and dynamics caused by the application of insecticides.

**Methodology**: We developed a population-genetic model of the spread of insecticide-resistance in a population of *Anopheles* vectors in response to insecticides used either as adulticides (focussing on insecticide-treated bed nets (ITNs)) or as larvicides (either for the control of malaria or, as an inadvertent side-product, in agriculture).

**Results**: We show that indoor use of insecticides leads to considerably less selection pressure than their use as larvicides, supporting the idea that most resistance of malaria vectors is due to the agricultural use of the insecticides that are also used for malaria control. The reasons for the relatively low selection pressure posed by adulticides are (i) that males are not affected by the ITNs and, in particular, (ii) that the insecticides are also repellents, keeping mosquitoes at bay from contacting the insecticide but also driving them to bite either people who do not use the insecticide or alternative hosts.

**Conclusion**: We conclude by discussing the opposing public health benefits of high repellency at an epidemiological and an evolutionary timescale: whereas repellency is beneficial to delay the evolution of resistance, other models have shown that it decreases the population-level protection of the insecticide.

## Abstract (precis)

Insecticides used for malaria control protect communities by killing larval or adult mosquitoes, and offer personal protection by repelling mosquitoes. We show that using the insecticides as larvicides selects for resistance more rapidly that using them against adult mosquitoes, and that their repellency can delay considerably the evolution of resistance.

## INTRODUCTION

Long-lasting insecticidal nets and indoor residual spraying (IRS) have dramatically reduced malaria transmission, for they protect users from being bitten by the mosquito vectors of malaria [1–4] and, by decreasing the longevity of mosquitoes, offer additional protection at the level of the community [5, 6]. Unfortunately this success is being eroded by the evolution of various mechanisms of resistance, including behavioural resistance (e.g. failure to be repelled or shifting from indoor-biting to outdoor-biting) [7, 8] and the focus of this article: insecticide-resistance (IR) rendering mosquitoes less sensitive to the insecticide used on the insecticide-treated bed nets (ITNs) [9, 10].

It is clear that the extensive use of insecticides for the control of malaria will increase the selection pressure on mosquitoes to evolve resistance. The problem is exacerbated by the fact that, after the sharp drop of the use of dichlorodiphenyltrichloroethane, vector control has become very reliant on a single class of insecticides, pyrethroids, which are also extensively used in other contexts, in particular agriculture. Widespread exposure of mosquitoes to agriculturally used insecticides, rather than exposure to ITNs, is indeed thought to be one of the main driving factors for the evolution of resistance [11] and therefore helps to undermine the efficiency of insecticide-based control measures. However, there is only limited understanding of the contribution of epidemiological, ecological and behavioural forces to the evolutionary dynamics of IR of malaria vectors.

A number of models have been developed to consider the role of these and other factors on the evolution of resistance. Barbosa and Hastings [12], e.g. use a population-genetic model to describe the rate of evolution when coverage of the bed nets is patchy, and predict the effect of using a chemical synergist to delay resistance. Extensions of similar approaches include age-specific effects of the insecticide to compare the effects of insecticides that are late-acting with those that lead to immediate death and to predict which mosquito life stage should be targeted [13–15].

A critical feature of these models is the ‘fitness’ of sensitive and resistant mosquitoes, which is described in various ways. The simplest one, e.g. Gourley *et al.* [13] assume that the insecticide increases the death rate of sensitive mosquitoes, but not of resistant ones, by a constant factor. Barbosa and Hastings [12] use a more complex formulation by including the proportion of houses that are covered by the bed nets. However, models that make mosquito fitness dependent on its behaviour and life-history provide significant advantages over others as they allow integration of knowledge of medical entomologists with the population genetics of the model. This approach has been followed by a number of authors [14–15].

For example, Koella *et al.* [16] combined a population-genetic approach with aspects of the mosquito’s feeding cycle to calculate ‘effective coverage’, the proportion of mosquitoes killed by the insecticide during a single gonotrophic cycle. We extended this approach by formulating a population-genetic model that calculates exposure rates from the mosquito’s feeding cycle similarly to the model described by Le Menach *et al.* [17]. In doing so, we propose behaviourally and epidemiologically based fitness functions that help us to understand more fully the predictions of the genetic model.

Our aim was to predict at least qualitatively the rate of evolution of IR under different transmission settings and under different characteristics of insecticide deployment and ITN interventions. We are in particular interested in (i) the relative selection pressures imposed by agriculturally used insecticides and ITNs and (ii) the effects of repellency and the tendency of mosquitoes to feed on non-human animals on the evolution of resistance. This model could hence contribute to inform recent campaigns that rely on the mass deployment of ITNs like the Roll Back Malaria initiative [18].

## METHODS

We assumed that IR is determined by a single gene with two alleles *R* and *S*, giving rise to three different genotypes: homozygote resistant individuals *RR*, homozygote sensitives *SS* and heterozygotes *RS*. We calculated the fitness of each genotype as its lifetime reproductive success, and used these in a standard population-genetic approach to predict the rate of change of the allele frequencies. We further assumed that insecticides can be used as adulticides on insecticide-treated nets and as larvicides, either in a direct attempt to control mosquitoes or as an inadvertent consequence of their agricultural use.

### Larvae

The survival of larvae is determined by the presence of the larvicide in a proportion *ψ* of the larval sites and by their resistance to the insecticide. We assume that sensitive mosquitoes are invariably killed by the insecticide, that mortality is reduced by the resistance *ρ* in *RR* individuals and that the resistance of heterozygous individuals is the product of *ρ* and the level of dominance *h*. If we standardize the model by assuming that all larvae in unexposed sites survive, the survival of sensitive individuals is 1−ψ, that of homozygously resistant individuals is 1−ψ(1−ρ) and that of heterozygous individuals is 1−ψ(1−hρ).

### Males

We assume that males never encounter the ITNs, so their fitness is determined only by larval survival and by a potential cost of resistance in fertility, *Z*. One potential mechanism of a male fertility cost could be via decreased competitiveness of resistant males for access to females [19]. We assume that dominance affects the cost of resistance identically to survival, so that the cost in heterozygotes is *h Z*. Thus the fitness of sensitive males is 1−ψ, that of resistant individuals is (1−ψ(1−ρ))(1−Z) and that of heterozygous individuals is (1−ψ(1−hρ))(1−hZ). (Table 1 for a summary of fitness measures).

**Table 1.** Fitnesses of male and female genotypes

Genotype	Males	Females
RR	$W_{m,RR}=(1-\psi \;(1-\rho ))\;(1-Z)$	$W_{f,RR}=\frac{\left(1-\psi \;(1-\rho ))\;\kappa \;(1-Z\right)}{1-(1-\mu_{gt})\;\frac{\sigma \;(1-Q\;\phi \;(1-(1-r)\;(s+\rho \;(1-s))))}{1-Q\;\phi \;r\;(1-\mu_{r})}}$
RS	$W_{m,RS}=(1-\psi \;(1-h\;\rho ))\;(1-h\;Z)$	$W_{f,RS}=\frac{\left(1-\psi \;(1-h\;\rho ))\;\kappa \;(1-h\;Z\right)}{1-(1-\mu_{gt})\;\frac{\sigma \;(1-Q\;\phi \;(1-(1-r)\;(s+h\;\rho \;(1-s))))}{1-Q\;\phi \;r\;(1-\mu_{r})}}$
SS	$W_{m,SS}=(1-\text{ψ})$	$W_{f,SS}=\frac{(1-\psi )\kappa }{1-(1-\mu_{gt})\;\frac{\sigma \;(1-Q\;\phi \;(1-(1-r)\;s))}{1-Q\;\phi \;r\;(1-\mu_{r})}}$

### Females

**Figure 1.**
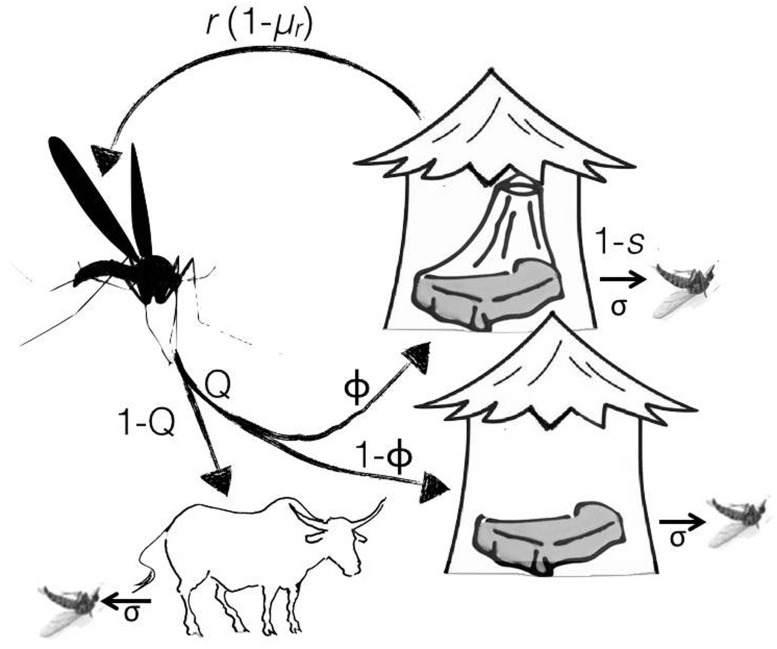
Host searching cycle of a mosquito: A mosquito bites an animal with probability 1−Q, while a proportion *Q* of the mosquitoes attempts to bite a human host inside houses, of which a proportion ϕ is protected by ITNs. We assume that mosquitoes survive feeding-associated death, same in humans and animals, with a probability *σ*. If mosquitoes target a protected house, there are three possible outcomes: the mosquito is repelled by the insecticide (or mechanically blocked by the net) with a probability *r*, or, if not repelled, it feeds and then escapes the risks of insecticide-associated death with probability *s*

We start by considering insecticide-sensitive mosquitoes. Once females have survived the insecticides in the larval sites to emerge as adults, their reproductive success is determined by the likelihood that they contact the insecticide on ITNs during their feeding attempts. To estimate this exposure rate, we modelled a mosquito feeding cycle as described in detail in [17] and reiterated in Fig. 1. Note that in this article, we are not interested in behavioural resistance, so that, in contrast to earlier articles, we ignore the possibility of outdoor feeding on humans. Although outdoor feeding would of course reduce the selection pressure for resistance it will not affect the qualitative conclusions of our model, providing that outdoor feeding has not directly evolved in response to the presence of ITNs.

We assume that at each feeding attempt, a proportion 1−Q of the mosquitoes feeds on an animal. A proportion *Q* of the mosquitoes attempt to enter a house to feed on a human. If the mosquito encounters a protected house (with probability ϕ), it is repelled (or mechanically blocked by the net) and starts a new host search with probability *r*. If it is not repelled (with probability 1−r), it survives the exposure to the insecticide with probability *s* and feeds successfully on the human host. We assume that each bite, be it on humans or animals, carries some risk of feeding-associated death, which the mosquito survives with a probability *σ*.

Overall, the probability that the mosquito obtains a blood meal and survives (i.e. is successful) during a single feeding attempt is

(1) $$\begin{array}{l} \sigma \;(1-Q)+\sigma \;Q\;(1-\phi )\\ +\sigma \;Q\;\phi \;(1-r)\;s=\sigma \;(1-Q\;\phi (1-(1-r)\;s)) \end{array}$$

Let us call this term Ξ. If the mosquito does not obtain a blood meal, it will start a new feeding attempt and repeat this until it is successful or dies. The mosquito is therefore successful, if it succeeds on its first attempt, or it is repelled once and succeeds on its second attempt, or it is repelled twice and then succeeds on its third attempt, etc. We assume that each time the mosquito is repelled and attempts to feed again, it will encounter an additional risk of death *μ_r_*. We can then calculate the probability of success as the geometric series:

(2) $$\begin{array}{l} \Xi +Q\phi r(1-\mu_{r})\Xi +{\left(Q\phi r(1-\mu_{r})\right)}^{2}\Xi +...\\ +{\left(Q\phi r(1-\mu_{r})\right)}^{n}\Xi +...\\ =\sigma \;(1-Q\;\phi \;(1-(1-r)\;s))\;\sum_{n=0}^{\infty }(Q\;\phi \;r\;(1-\mu_{r})\;{)}^{n}\\ =\frac{\sigma \;(1-Q\;\phi \;(1-(1-r)\;s))}{1-Q\;\phi \;r\;(1-\mu_{r})} \end{array}$$

Once fed, the mosquito must survive through the duration of its gonotrophic cycle (i.e. the time it takes to develop and lay its eggs) before it starts a new feeding attempt. The probability of feeding-independent mortality during the gonotrophic cycle is $\mu_{gt}=1-{\left(1-\mu \right)}^{gt}$, where *μ* is the daily mortality and *gt* is the length of the gonotrophic cycle. Note that, in contrast to [17], we assume that the length of the gonotrophic cycle is not modified by repeated host searches. This is a good approximation unless each feeding attempt lasts a long time. The latter could happen, e.g. if a mosquito has to travel large distances between potential hosts or if ITN coverage is close to 100%. This can be understood by considering a situation of high host density; here, the search for a new host may last only for a few minutes. Then, even if the mosquito is repelled 10 times (e.g. under conditions of say 95% coverage and 9% repellency), the gonotrophic cycle length will at most increase by a few percent. The probability of surviving a gonotrophic cycle (the combination of feeding-related and feeding-independent mortality) is hence:

(3) $$(1-\mu_{gt})\;\frac{\sigma \;(1-Q\;\phi \;(1-(1-r)\;s))}{1-Q\;\phi \;r\;(1-\mu_{r})}$$

giving an average lifespan (in multiples of the gonotrophic cycle) of:

(4) $$\frac{1}{1-(1-\mu_{gt})\;\frac{\sigma \;(1-Q\;\phi \;(1-(1-r)\;s))}{1-Q\;\phi \;r\;(1-\mu_{r})}}$$

IR affects the probability that a mosquito survives blood feeding once it has entered a house. The parameter *s* in equation 4 is the probability that sensitive mosquitoes survive exposure to the insecticide. In homozygously resistant mosquitoes, the sensitivity to the insecticide is reduced by the parameter *ρ*, so that the probability of being killed by the insecticide is reduced to (1−s)(1−ρ) and the probability of success inside an ITN-home is (s+ρ (1−s)); the probability that heterozygous mosquitoes succeed is (s+h ρ (1−s)).

Using these survival terms in equation 2, we obtained the average longevity of each genotype of adult female mosquitoes:

(5) $$\text{Lifespan (}SS\text{)}=\frac{1}{1-(1-\mu_{gt})\;\frac{\sigma \;(1-Q\;\phi \;(1-(1-r)\;s))}{1-Q\;\phi \;r\;(1-\mu_{r})}}$$

(6) $$\text{Lifespan (}RS\text{)}=\frac{1}{1-(1-\mu_{gt})\;\frac{\sigma \;(1-Q\;\phi \;(1-(1-r)\;(s+h\;\rho \;(1-s))))}{1-Q\;\phi \;r\;(1-\mu_{r})}}$$

(7) $$\text{Lifespan (}RR\text{) }=\frac{1}{1-(1-\mu_{gt})\;\frac{\sigma \;(1-Q\;\phi \;(1-(1-r)\;(s+\rho \;(1-s))))}{1-Q\;\phi \;r\;(1-\mu_{r})}}$$

The fitness of each genotype is obtained by multiplying this quantity by a typical value of female mosquito fertility, *κ*, and by the probability that larvae survive the insecticide applied to larval sites, 1−ψ. Larval mortality is affected by resistance according to the equations given earlier for the mortality of males. We finally assume that resistance is costly in that the fecundity of homozygous resistant mosquitoes is reduced by the factor *Z* and that of heterozygotes is reduced by h Z (note that for simplicity, we assume that the cost of resistance is equal for males and for females). This gives the fitness values of males and females, shown in Table 1.

### Evolution

Designating the frequencies of the resistance allele in males and in females by *p_m_* and *p_f_*, respectively, and the frequencies of the susceptibility allele by $q_{m}=1-p_{m}$ and $q_{f}=1-p_{f}$, the genotype frequencies in males and females after selection are given by the following equations [20]:

$$SS_{m}=\frac{W_{m,SS}\;q_{m}\;q_{f}}{{\bar{W}}_{m}}$$

$$RS_{m}=\frac{W_{m,RS}\;(p_{m}\;q_{f}+p_{f}\;q_{m})}{{\bar{W}}_{m}}$$

(8) $$RR_{m}=\frac{W_{m,RR}\;p_{m}\;p_{f}}{{\bar{W}}_{m}}$$

$$SS_{f}=\frac{W_{f,SS}\;q_{m}\;q_{f}}{{\bar{W}}_{f}}$$

$$RS_{f}=\frac{W_{f,RS}\;(p_{m}\;q_{f}+p_{f}\;q_{m})}{{\bar{W}}_{f}}$$

$$RR_{f}=\frac{W_{f,RR}\;p_{m}\;p_{f}}{{\bar{W}}_{f}}$$

where ${\bar{W}}_{M}$ and ${\bar{W}}_{F}$ are the mean fitnesses of males and females in the population and are given by:

(9) $${\bar{W}}_{m}=W_{m,RR}\;p_{m}p_{f}+W_{m,RS}\;(p_{m}\;q_{f}+p_{f}\;q_{m})+W_{m,SS}\;q_{m}\;q_{f}$$

(10) $${\bar{W}}_{f}=W_{f,RR}\;p_{m}p_{f}+W_{f,RS}\;(p_{m}\;q_{f}+p_{f}\;q_{m})+W_{f,SS}\;q_{m}\;q_{f}$$

We assume discrete and non-overlapping mosquito generations. Consequently, the frequencies of the resistance allele in males and females from one mosquito (parental) generation, (*t*), to the next (offspring) generation, (t+1), are:

(11) $$\begin{array}{c} p_{m}(t+1)=\\ \frac{W_{m,RR}\;p_{m}(t)\;p_{f}(t)+0.5\;W_{m,RS}\;(p_{m}(t)\;q_{f}(t)+p_{f}(t)\;q_{m}(t))}{{\bar{W}}_{m}} \end{array}$$

(12) $$\begin{array}{c} p_{f}(t+1)=\\ \frac{W_{f,RR}\;p_{f}(t)\;p_{m}(t)+0.5\;W_{f,RS}\;(p_{f}(t)\;q_{m}(t)+p_{m}(t)\;q_{f}(t))}{{\bar{W}}_{f}} \end{array}$$

The parameters of our model, together with their typical values, are listed in Table 2.

**Table 2.** Parameters and their typical values

Parameter	Explanation	Typical value	Reference
ϕ	ITN coverage		
*Ψ*	proportion of mosquitoes exposed		
	to agriculturally used insecticide		
*Q*	feeding rate on humans	0.7	[21]
*r*	repellency rate	0.7	[22]
*s*	probability of surviving ITN insecticide exposure	0.16	[22]
*Σ*	survival of risk of feeding-induced death	0.9	
*gt*	length of gonotrophic cycle (days)	3	[23]
*Μ*	daily mortality rate of vector	0.1	[24]
*μ_gt_*	mortality in one gonotrophic cycle	0.27	[24]
*μ_r_*	additional mortality if repelled once	0.03	
*h*	dominance of IR allele	0.25	[25]
*ρ*	level of resistance conferred by IR allele	0.95	
*Z*	cost of resistance	0.10	[26]
*κ*	female fecundity	100	[27]

## RESULTS

**Figure 2.**
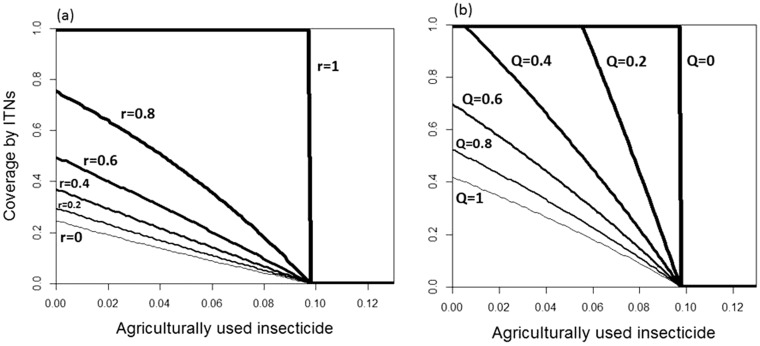
The combination of coverage by ITNs and by larvicides that enable resistance to be fixed (lines) or eliminated (below lines) for (**a**) repellency, *r* ranging from 0 along the thin line to 1 along the thick line with an interval of 0.2 between adjacent lines and for (**b**) human feeding, *Q*, ranging from 1 along the thin line to 0 along the thick line. Other parameter values are given in Table 2

**Figure 3.**
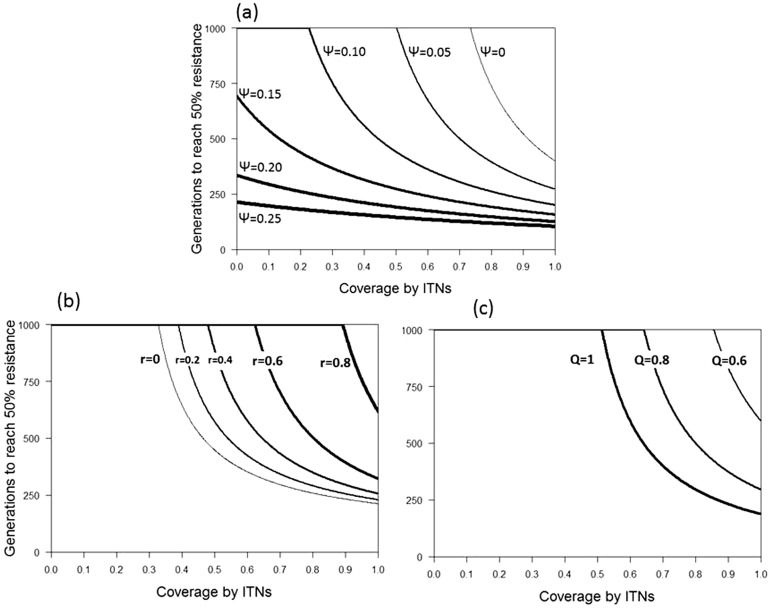
The rate of evolution of resistance against ITNs as a function of their coverage and for several parameter values. The rate of evolution is given as the time (in number of mosquito generations) it takes for a resistance allele to reach a frequency of 50%, starting with a frequency of 1/100000, (**a**) The role of a larvicide used simultaneously at a coverage *ψ* ranging from 0 (thin line) to 25% (thick line). (**b**) The role of the repellency of the ITN, with repellency *r* ranging from 0 (thin line) to 0.8 (thick line). (**c**) The role of the likelihood that mosquitoes feed on animals, with indoor human-feeding *Q* ranging from 1 (thick line) to 0.6 (thin line). Other parameter values are given in Table 2

Our model always leads to either fixation or elimination of the resistance allele. We therefore show two types of results, obtained from simulations: (i) the conditions that lead to fixation of the allele (Fig. 2) and (ii), for conditions that enable fixation of resistance, the number of generations it takes for the allele to reach a frequency of 50% (Fig. 3).

We considered two pressures selecting for IR: ITNs, to which only adult females are exposed, and larvicides, which affect larvae of both sexes. Figure 2 shows that the selection pressure imposed by ITNs is considerably weaker than that imposed by the larvicides. Indeed, with the typical parameters given in Table 2, resistance is fixed as a response to only larvicides if more than 10% of the larval sites are treated with a lethal concentration of the insecticide, whereas if mosquitoes are exposed to ITNs only, resistance is fixed only if at least ∼20% of the houses are treated with ITNs even in the extreme case of no repellency (Fig. 2a) and no animal-feeding (Fig. 2b). The selection pressure due to ITNs depends strongly on the repellency of the insecticide and the extent of animal-feeding by the mosquitoes. As repellency increases, more mosquitoes are diverted from the insecticide, so that it becomes less likely that resistance is fixed; if all mosquitoes are repelled, the insecticide kills no mosquitoes, so the ITNs impose no selection for resistance (Fig. 2a). Similarly, if mosquitoes are more likely to feed on animals, they are less exposed to the insecticide, so that the selection pressure decreases (Fig. 2b).

These results are reflected in simulations giving the time it takes for the resistance allele to reach a frequency of 50% (Fig. 3), starting at an initial gene frequency of $p_{f}=p_{m}=0.00001$. In the absence of larvicides, the time to evolve resistance decreases strongly with increasing coverage by ITNs (Fig. 3a). However, as the coverage of larvicides increases, the effect of coverage by ITNs on the time to evolve resistance diminishes. Indeed, at high coverage by the larvicides, the effect of ITNs is almost negligible, whereas even at complete coverage by ITNs increasing the use of larvicides substantially decreases the time to evolve resistance (Fig. 3a). The time to evolve resistance is also strongly increased by the repellency of the insecticide (Fig. 3b) and the likelihood that mosquitoes feed on animals (Fig. 3c).

**Figure 4.**
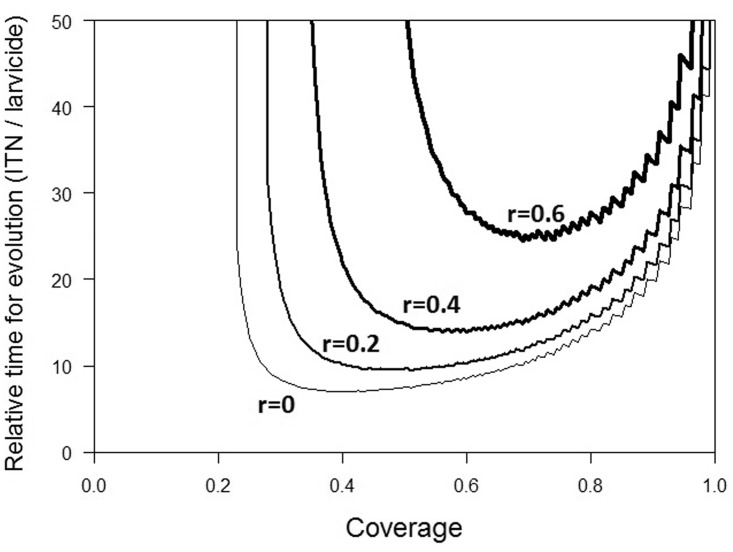
The comparison of the rate of evolution in situations where the insecticide is used only on ITNs or only as larvicides. The y-axis shows the ratio of the two times (in number of mosquito generations) it takes for a resistance allele to reach a frequency of 50%, starting with a frequency of 0.00001 in males and females; the x-axis shows the coverage by the insecticide in either situation. The lines show different levels of repellency, *r*, ranging from 0 (thin line) to 0.6 (thick line). Other parameter values are given in Table 2

The difference between the selection pressures posed by larvicides and by ITNs is seen most clearly in Fig. 4, which shows the ratio of the time it takes for the resistance allele to reach a frequency of 50% in situations where an insecticide is used only on an ITN or as a larvicide with the same level of coverage. Larvicides have the strongest effect on driving the evolution of IR with speeding up the evolution at least 8–10 times compared with a same coverage of ITNs alone. This effect however also depends strongly on the repellency of the net: higher levels of repellency slow significantly the evolution of resistance.

## DISCUSSION

Our model, which adds the behaviour of mosquitoes to population-genetic theory, shows that ITNs can lead to a substantial selection pressure for the evolution of IR. Yet, this selection pressure is weakened considerably by the repellent effects of the insecticide and, in some ecological settings, by the propensity of mosquitoes to feed on animals other than humans. Furthermore, the selection pressure imposed by larvicides is considerably stronger than that imposed by ITNs.

With the typical parameters (Table 2), we found in reviews of field studies (e.g. 70% repellency and 70% human-feeding), our model predicts that for intermediate to high coverage by ITNs it takes ∼200–300 mosquito generations for the frequency of a resistance allele to reach 50%. If we assume a year-round transmission setting with the mosquito’s generation time of ∼20 days, that would translate into a time of between 10 and 15 years. Allowing for a considerable variation in the values of transmission parameters, this timescale is roughly similar to what is observed in reality. There is much evidence that the deployment of ITN or IRS fuels the rapid spread of resistance alleles like the kdr allele [28–31]. In a controlled field trial in Mexico, e.g. *Anopheles* populations went from 0 to 20% resistance in 3 years of IRS [32], and once close to complete coverage by ITNs was started in Western Kenya most mosquitoes were resistant within 10 years [33].

Nevertheless, it appears that it is often agricultural use of larvicides rather than malaria control that underlies the evolution of resistance in *Anopheline* mosquitoes [11, 34–36]. Our model gives a theoretical backing to this observation. Indeed, our model predicts that for a wide range of parameter values it takes at least 20 times longer for resistance to evolve if ITNs are the sole selection pressure than if larvicides are (Fig. 4). This should come as no surprise: larvicides impose stronger pressure than ITNs, for they target all individuals, whereas ITNs target only females. Rather than comparing ‘coverage’ of both intervention strategies as defined in this article, it would be a fruitful effort to compare the effect on resistance evolution of those interventions employing different bases of comparison, e.g. comparing the effect of a certain quantity of insecticide used either as a larvicide or an ITN. This could for example take the shape of a cost-effectiveness analyses (cost-‘resistance’ analysis), similar to efficiency analyses run for antimalarial intervention methods [37, 38]. Finally, it has to be recognized however that the importance of the larvicides, whether deliberately deployed for mosquito control (larval source management) or as an agrochemical by-product, is highly dependent on mosquito control or agricultural activity in the considered region [39, 40] and that both repellency and animal-feeding keep mosquitoes away from the ITNs and therefore reduce their exposure to the lethal effects of the insecticide.

Naturally, the quantitative predictions of our model depend strongly on its assumptions. Several of these are reasonable. We assume, e.g. that the resistance allele gives a similar level of resistance to larvae and adults and that the level of dominance is similar in the two life stages, as observed in insecticide bioassays conducted with larval and adult mosquitoes of various genotypes [41, 42]. In our model, we talk about a lethal concentration but in the natural setting, this will also depend on the exposure length (compare a short contact with an ITN to a more prolonged contact in the larval environment) as well as the concentration of the insecticide in a given environment (potentially a stronger concentration on an ITN).

Other assumptions make little difference to the conclusion. Thus, we assume that the cost of resistance is paid through reduced fecundity rather than through reduced longevity, for which there is some experimental evidence [16, 43]. We avoided doing so in order not to further complicate the expression for longevity. We also assume that males experience a similar cost of resistance that affects fertility. This could for example happen via reduced competitive success for females compared with susceptible males, reduced sperm viability or female preference for susceptible males (either via standard or cryptic sexual selection). Some evidence for a male fertility cost of resistance, if in competition with susceptible males, has been provided by [19] for *Culex pipiens*, but we are unaware of any investigation that has looked for a male cost in *Anopheles* mosquitoes. A main assumption is that the behaviour of the mosquitoes—the likelihoods that they bite animals and that are repelled by insecticides—does not evolve as a response to insecticide pressure. Any genetic variation would of course lead to selection pressure, as the mosquitoes would thereby be less likely to be killed [8]. The qualitative consequences of selection for behavioural resistance for the evolution of IR seem clear. In the simplest case, when behaviour is not linked to resistance, selection would reduce contact with the insecticide, thus weakening the selection pressure for true resistance and strengthening our conclusion that larvicides impose stronger selection for resistance than ITNs. Things become more complicated if behaviour and resistance are genetically linked. In this case the evolutionary dynamics will depend critically on the sign of the genetic correlation between behaviour and resistance—a positive correlation would enforce selection of resistance; a negative one would constrain it. As we have no evidence of such a correlation and can therefore not make more quantitative predictions, we ignore behavioural resistance in our model.

An important feature of our model is that it uses the mosquitoes’ behaviour to estimate their fitness, and thus combines an ecological approach with population genetics. The importance of the behaviour linked to the repellency of the ITNs is clearly seen in Figs. 2a and 3b. Most other models describing the evolution of IR, whether discussing the mosquitoes that transmit malaria [12, 44] or other insects [45] ignore the behavioural response of the insects to the insecticide. On the other hand, several epidemiological models have profited from incorporating the mosquitoes’ behaviour, thus emphasizing the importance of linking behavioural ecology with the epidemiology and evolution of resistance [15, 16] ([46] for an application to behavioural resistance).

In summary, we described a scenario in which IR could evolve in response to a given coverage by ITNs. First, we showed that, while ITNs can lead to the rapid evolution of resistance, larvicides—whether they are used for malaria control or kill mosquitoes as a by-product of agricultural use—are likely to impose a much stronger selection pressure. This gives the theoretical basis for the claim that it is the agricultural use of insecticides rather than ITNs that has driven the evolution of insecticide-resistant malaria vectors in many parts of Africa. Second, we showed that the repellent property of ITNs has a strong effect on the evolution of IR, so that the strong repellency can help to maintain the efficacy of insecticides in the long-term. This benefit to the community complicates the conflicting effects of repellency, which on the one hand offers personal protection to their users [47] but on the other hand may have little impact [48] or can even have detrimental effects on the community as a whole by keeping mosquitoes from being killed and therefore increasing prevalence [49] (P. L. G. Birget and J. C. Koella, submitted for publication). Overall, thus, attempts to slow the evolution of resistance against insecticides must take into account the complexity of the evolutionary process, which is substantially influenced by details of the use of insecticides and of the mosquitoes’ behavioural response to the insecticide.

## Supplementary Material

Supplementary Data
