# Supplementary material for: A genetic model of the effects of insecticide-treated bed nets on the evolution of insecticide-resistance
Source: Evol Med Public Health. 2015 Aug 29;2015(1):205–15. doi: 10.1093/emph/eov019 (PMC4571732; doi:10.1093/emph/eov019)
Supplement: Supplementary Data [file supp_2015_1_205__index.html]

A genetic model of the effects of insecticide-treated bed nets on the evolution of insecticide-resistance — Supplementary Data 

# A genetic model of the effects of insecticide-treated bed nets on the evolution of insecticide-resistance

## Supplementary Data

files

- Supplementary Data - zip file
