# Supplementary figures and images for: A genetic model of the effects of insecticide-treated bed nets on the evolution of insecticide-resistance
Source: Evol Med Public Health. 2015 Aug 29;2015(1):205–15. doi: 10.1093/emph/eov019 (PMC4571732; doi:10.1093/emph/eov019)

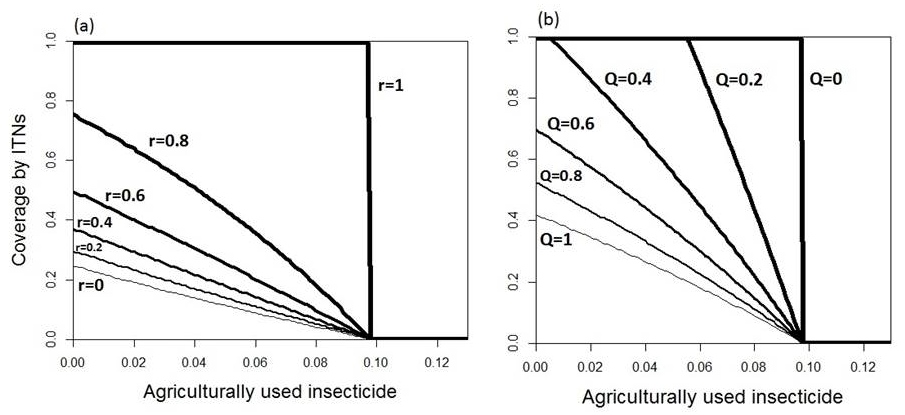

Supplement: Supplementary Data [file supp_eov019_suppl_data.zip › SmallFixFig.jpg]

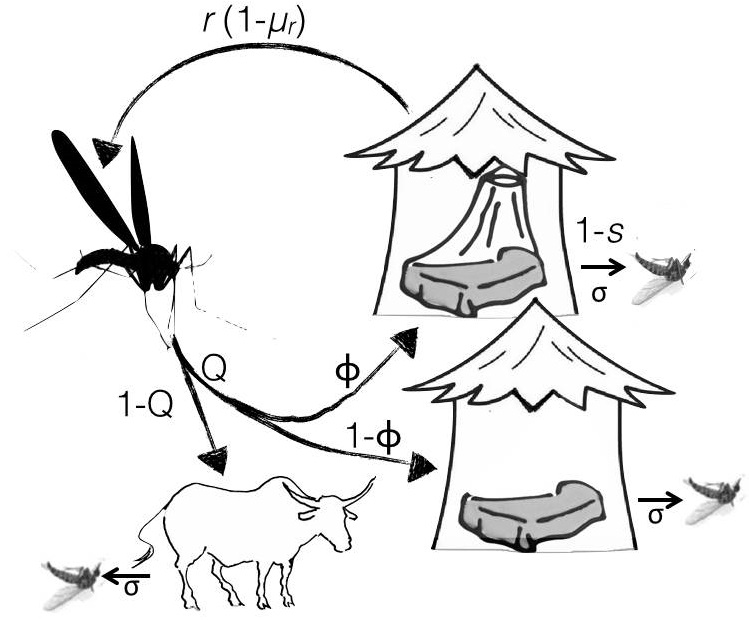

Supplement: Supplementary Data [file supp_eov019_suppl_data.zip › alteredFeedCycle.jpg]

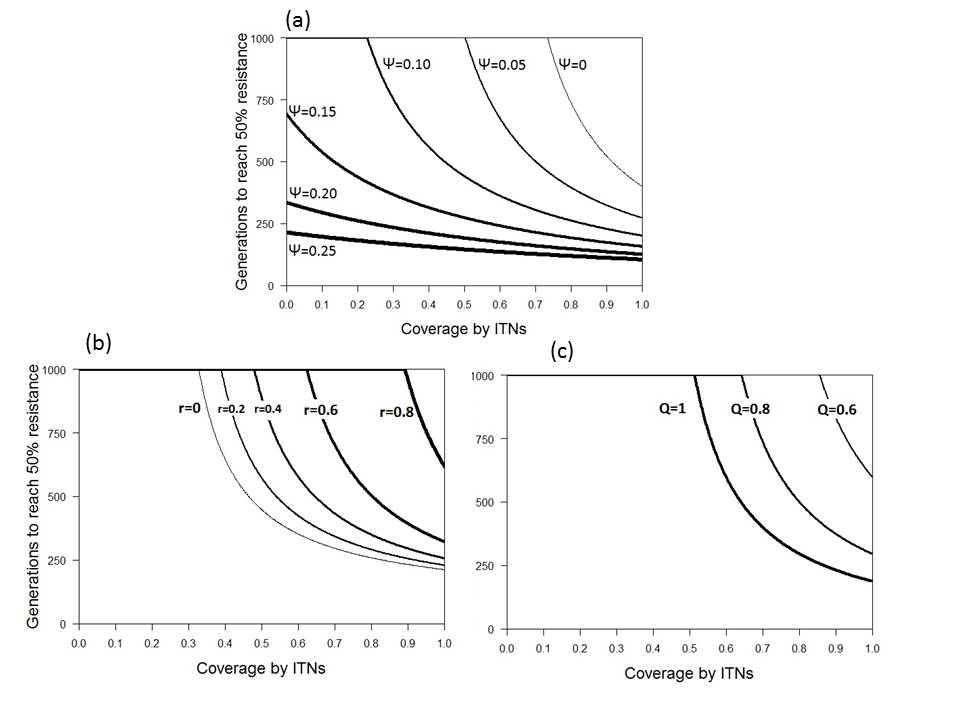

Supplement: Supplementary Data [file supp_eov019_suppl_data.zip › BigFixFig.jpg]

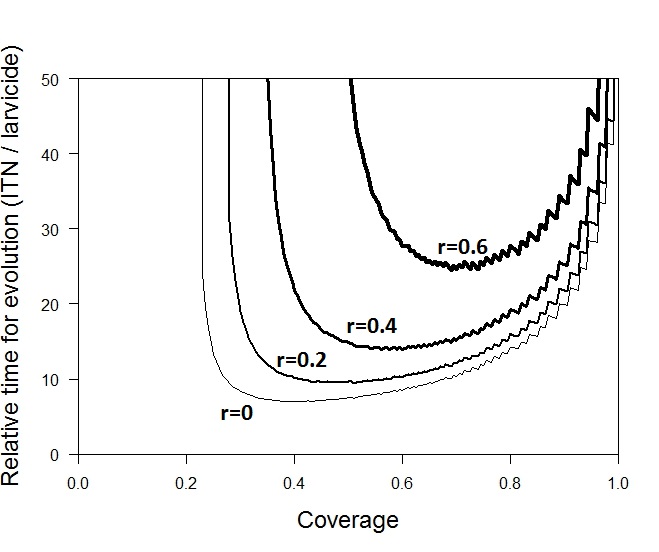

Supplement: Supplementary Data [file supp_eov019_suppl_data.zip › ITNvsAgri.jpg]
